# Opinion Dynamics with Higher-Order Bounded Confidence

**DOI:** 10.3390/e24091300

**Published:** 2022-09-14

**Authors:** Chaoqian Wang

**Affiliations:** Program for Computational Social Science, Department of Computational and Data Sciences, George Mason University, Fairfax, VA 22030, USA; CqWang814921147@outlook.com or cwang50@gmu.edu

**Keywords:** opinion dynamics, bounded confidence, higher-order interaction, HK model

## Abstract

The higher-order interactions in complex systems are gaining attention. Extending the classic bounded confidence model where an agent’s opinion update is the average opinion of its peers, this paper proposes a higher-order version of the bounded confidence model. Each agent organizes a group opinion discussion among its peers. Then, the discussion’s result influences all participants’ opinions. Since an agent is also the peer of its peers, the agent actually participates in multiple group discussions. We assume the agent’s opinion update is the average over multiple group discussions. The opinion dynamics rules can be arbitrary in each discussion. In this work, we experiment with two discussion rules: centralized and decentralized. We show that the centralized rule is equivalent to the classic bounded confidence model. The decentralized rule, however, can promote opinion consensus. In need of modeling specific real-life scenarios, the higher-order bounded confidence is more convenient to combine with other higher-order interactions, from the contagion process to evolutionary dynamics.

## 1. Introduction

Opinion dynamics, being one of the essential branches of sociophysics, studies the statistical physics of collective opinion evolution driven by microscopic rules of individuals [1]. Opinion dynamics models can be broadly classified into two categories concerning the opinion space [2]: the discrete opinion space [3,4,5,6,7,8,9,10,11], and the continuous opinion space [12,13,14,15,16]. The models based on discrete opinion space usually assume two opposing opinions in the system (e.g., +1, −1, or A, B, etc.). The classic discrete opinion dynamics models include the voter model [3,4,5], the Sznajd model [6,7,8], and the Galam model [9,10,11]. Another class of models is based on continuous opinion space, where an individual’s opinion is measured by a real number between 0 and 1, inclusive. One of the most classic models with continuous opinion space is the DeGrootian model [2,12,13,14]. Then, it was not until researchers introduced the bounded confidence into the continuous opinion dynamics that the well-known Deffuant–Weisbuch (DW) model [15] and Hegselmann–Krause (HK) model [16] were born. The HK model can be considered a mean-field approximation to the DW model. Although both the DW and HK models are based on bounded confidence, we only focus on the HK model in this work.

The HK model assumes that an agent (i.e., an individual) only accepts opinions that do not differ from its own by more than a critical value. This critical value is labeled as the bounded confidence. This work denotes the bounded confidence by *r* (r≥0) and supposes there are *N* agents in a well-mixed population. The opinion of agent *i* at time step *t* is denoted by $x_{i}\left(t\right)$. In the classic HK model, an agent’s opinion update is the average of all acceptable opinions:

(1) $$x_{i}\left(t+1\right)=\frac{1}{|N_{i}\left(t\right)|}\sum_{j\in N_{i}\left(t\right)}x_{j}\left(t\right),$$

where $N_{i}\left(t\right)=\left\{j|\right|x_{i}\left(t\right)-x_{j}\left(t\right)\left|\leq r,\;j=1,2,\cdots ,N\right\}$ and $|N_{i}\left(t\right)|$ is the number of elements in set $N_{i}\left(t\right)$. Note that self-loop j=i is allowed. The opinion updates of all agents are synchronous. Letting the system evolve according to Equation (1), we can obtain a stability (i.e., stationary) opinion profile. The opinion profile switches from consensus to polarization and fragmentation as the bounded confidence *r* decreases, intuitively elucidating the so-called “information cocoon” [17] where individuals are bound to a cluster of similar opinions and do not interact with other clusters.

A variety of mathematical tools have been used to investigate the properties of the bounded confidence model, by which the convergence [18,19], the pattern formation [20], the entropy [21], and the control theory [22] in the bounded confidence model have been studied. Other works focus on innovations in the model itself. Some of them introduced various new factors [23,24,25,26,27,28,29,30,31,32,33], such as the opinion leader [23], the memory [24], the expression and private opinion [25], the fuzzy inference [26], the stubbornness [27,28,29], and the noise [23,30,31,32,33], to the classic bounded confidence model. Others consider different possibilities of evolutionary mechanisms of the system [27,34,35,36,37,38,39], such as the heterogeneous bounded confidence [27,34,35], the heterogeneous pressure [36,37], and the circular opinion space [38,39]. One of the most important topics in the bounded confidence model is how to promote the opinion consensus. In this regard, some works have investigated the conditions of consensus formation [40,41,42,43]. Other works introduced new factors or mechanisms, such as the external activation [44], and the combination of pairwise and group interactions [45], with the aim of promoting opinion consensus.

As we mentioned previously, in the classic bounded confidence model, an agent’s opinion update is directly the average over its peers. In other words, in the framework of the classic bounded confidence model, it is not straightforward to consider higher-order interactions. This is an important entry point, since higher-order interactions beyond pairwise ones can model real-life scenarios in a more intuitive way and have been revealed for non-trivial phenomena that do not exist in pairwise interactions [46,47,48]. With these attractive advantages, higher-order interactions have been introduced into a wide range of complex systems, from contagion process [49,50,51] to evolutionary games [52,53,54], by means of hypergraphs or simplicial complexes. In particular, opinion dynamics based on higher-order interactions have sprouted [55,56,57,58]. Neuhäuser et al. [55] studied opinion consensus dynamics by multibody interactions and found that the resulting dynamics can cause shifts away from the average system state. Sahasrabuddhe et al. [56] further explored consensus dynamics on hypergraphs based on sociological theories and investigated relevant dynamics on real-world structures. Hickok et al. [57] studied the Deffuant–Weisbuch bounded confidence model on hypergraphs and found that agents can jump from one opinion cluster to another in a single time step, which is impossible in bounded confidence models with pairwise interactions. In addition, Horstmeyer and Kuehn [58] investigated a coevolutionary voter model on simplicial complexes.

The work mentioned above on opinion dynamics was carried out on hypergraphs or simplicial complexes in a strict way but did not relate the concept of higher-order interactions to the bounded confidence directly. The theoretical concept of “higher-order bounded confidence” has corresponding realistic scenarios; for example, when opinion discussions can happen among a group of people instead of two-by-two, a person may want to join in a discussion because her opinion is close to the discussion’s organizer. As a result, she is involved in the group opinion discussion even if the opinions of some participants are not close to her.

**Figure 1:**
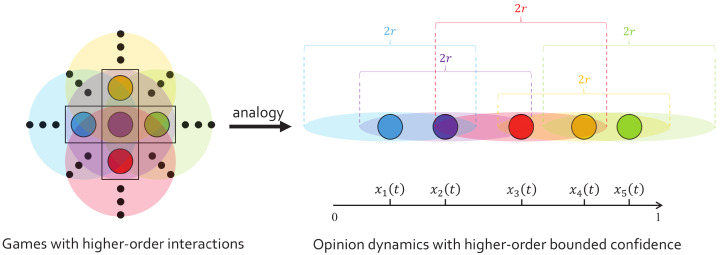
Schematic of the analogy, from games with higher-order interactions (**left**), to opinion dynamics with higher-order bounded confidence (**right**). (**left**) five agents on a regular square lattice. The purple agent organizes a multiplayer game among the five agents (its nearest neighbors and itself), while also participates in the games organized by the other four agents. (**right**) five agents on a continuous one-dimensional opinion space. The purple agent organizes a group opinion discussion among the blue, purple, and red agents within its bounded confidence, while also participates in the discussions organized by the blue and red agents.

In this way, considering both theoretical and practical importance, this work tries to provide the introduction of higher-order bounded confidence at a theoretical model level. Similar algorithms can be found in many previous multidisciplinary fields, but let us employ a simple one to analog, the multiplayer evolutionary games (e.g., the public goods game [59]). In multiplayer games, each focal agent organizes a game among its neighbors and itself. Meanwhile, its neighbors also perform the same action. As a result, each agent actually participates in multiple games organized by its neighbors and itself (see Figure 1, left). In this regard, the common algorithm is to average the results obtained by these multiple games. In this work, we analog this algorithm to the bounded confidence model. While the multiplayer games are based on constant interactions, the peers that an agent interacts with in the bounded confidence model are determined by the opinion distance, which varies at each time step. Here, the homogeneity of bounded confidence ensures that the “peer interaction” is always undirected (i.e., interactions are always mutual, see Figure 1, right). Therefore, we can perform the following analogous migration of the higher-order interaction algorithm. First, each agent organizes a group opinion discussion among its peers. Second, since the peers perform the same action, each agent participates in multiple opinion discussions organized by its peers. Finally, the opinion update of an agent is the average over the results obtained from these multiple discussions.

The structure of this paper is described below. While the rules followed by a single group opinion discussion could be arbitrary, Section 2 gives two basic rules: centralized and decentralized. The former is equivalent to the classic HK model, while the latter leads to “higher-order” interactions. In Section 3, we explore the role of decentralized discussion in promoting the opinion consensus, compared to the classic HK model. In Section 4, we review the higher-order bounded confidence framework and discuss potential future development.

## 2. Model

Consider a well-mixed population of *N* agents. At time step *t*, each agent i=1,2,⋯,N holds an opinion $x_{i}\left(t\right)$. Suppose the opinion is represented by a continuous real number between 0 and 1: $0\leq x_{i}\left(t\right)\leq 1$. For each agent, we denote a peer set $N_{i}\left(t\right)=\left\{j|\right|x_{i}\left(t\right)-x_{j}\left(t\right)\left|\leq r,\;j=1,2,\cdots ,N\right\}$, where *r* represents the bounded confidence. We assume an agent *i* only interacts with its peer agents in $N_{i}\left(t\right)$, whose opinions are not more than *r* away from agent *i*. We allow self-loop: $i\in N_{i}\left(t\right)$.

The interactions are second-order. At time step *t*, we go through the *N* agents. Each focal agent *i* organizes a group opinion discussion among its peers $j\in N_{i}\left(t\right)$. The opinions of all participants $x_{j}\left(t\right)$ can influence the discussion’s outcome. We denote the discussion’s outcome by $o_{i}\left(t\right)$. The *N* agents organize their discussions synchronously.

Note that an agent is also the peer of its peers. In this way, an agent *i* should participate in $|N_{i}\left(t\right)|$ discussions at each time step, where $|N_{i}\left(t\right)|$ denotes the number of elements in $N_{i}\left(t\right)$. We assume each discussion works in the opinion updates of all participants, and the opinion update of each agent is the average over all discussions it participates in. That is, for an agent *i*, the opinion update is

(2) $$x_{i}\left(t+1\right)=\frac{1}{|N_{i}\left(t\right)|}\sum_{j\in N_{i}\left(t\right)}o_{j}\left(t\right).$$

The *N* agents update their opinions synchronously. Unlike Equation (1), where $x_{j}\left(t\right)$ is the outcome of pairwise interaction, in Equation (2), $o_{j}\left(t\right)$ is the outcome of group interactions.

Next, we further give $o_{j}\left(t\right)$ concrete forms. For example, Equation (2) degenerates to the classic HK model, if we give $o_{j}\left(t\right)=x_{j}\left(t\right)$. In this case, the discussion organized by agent *j* is “centralized” because the organizer *j* directly adopts its own opinion as the discussion’s outcome. This is reminiscent of “stubbornness” [27,28,29] with which agents do not change opinions. However, the centralized rule here simply means the organizer’s opinion is the outcome of a group discussion.

Other than the centralized rule, let us propose another rule—the “decentralized”. Literally, if the discussion is decentralized, the discussion’s outcome is the average opinion over all participants. As a possible result, the outcome $o_{j}\left(t\right)$ centered on agent *j* whose opinion is initially at a distance $|x_{j}\left(t\right)-x_{i}\left(t\right)|<r$ from agent *i* could fall outside the interaction range (i.e., $|o_{j}\left(t\right)-x_{i}\left(t\right)|>r$). However, *i* still interacts with *j*, which does not happen in the classic HK model. To sum up,

(3) $$o_{j}\left(t\right)=\left\{\begin{array}{cc} \; & x_{j}\left(t\right),\;\;\;\;\;\mathrm{if}\;\mathrm{agent}\;j\;\mathrm{is}\;\mathrm{centralized},\\ \; & \frac{1}{|N_{j}\left(t\right)|}\sum_{k\in N_{j}\left(t\right)}x_{k}\left(t\right),\;\;\;\;\;\mathrm{if}\;\mathrm{agent}\;j\;\mathrm{is}\;\mathrm{decentralized}. \end{array}\right.$$

We classify agent types by centralized and decentralized, who only organize centralized and decentralized discussions, respectively. We denote the fraction of decentralized agents in the population by α (0≤α≤1), while 1−α is the fraction of centralized agents. The type of an agent does not change with time.

The decentralized rule may allow agents to interact at an opinion distance larger than *r*, which cannot happen in the classic HK model. For instance, we take the purple agent on the right side of Figure 1, whose opinion is denoted by $x_{2}\left(t\right)$ according to the schematic. If we assume that the blue agent is “centralized,” and the purple and the red agents are “decentralized,” then $x_{2}\left(t+1\right)=\left[o_{1}\left(t\right)+o_{2}\left(t\right)+o_{3}\left(t\right)\right]/3$, where $o_{1}\left(t\right)=x_{1}\left(t\right)$, $o_{2}\left(t\right)=\left[x_{1}\left(t\right)+x_{2}\left(t\right)+x_{3}\left(t\right)\right]/3$, $o_{3}\left(t\right)=\left[x_{2}\left(t\right)+x_{3}\left(t\right)+x_{4}\left(t\right)\right]/3$. Thus, in this example, we see that the purple agent interacts with the orange agent at an opinion distance larger than *r*.

It might also be necessary to mention that a single outcome’s concrete form $o_{j}\left(t\right)$ could be arbitrary, not limited to the “centralized” or “decentralized” adopted in this paper, as long as it is a function of the opinions of *j*’s peers *k*, $o_{j}\left(t\right)=f\left(x_{k}\left(t\right)|k\in N_{j}\left(t\right)\right)$.

## 3. Numerical Simulation

### 3.1. Experiment Design

In the simulation, we fix N=1000. At t=0, we set each agent’s initial opinion $x_{i}\left(0\right)$ uniformly at random between 0 and 1, inclusive. Among the *N* agents, the decentralized agents totaling αN are randomly designated, and the remaining (1−α)N are centralized. Then, we simulate the system according to the rules established in Section 2.

**Figure 2:**
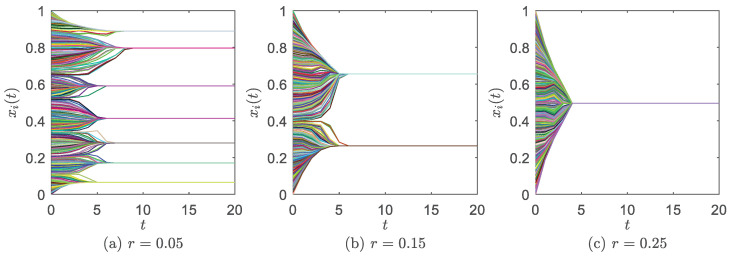
Each agent’s opinion $x_{i}\left(t\right)$, i=1,2,⋯,N, as a function of time *t* at α=1 and different *r*. (**a**) r=0.05. (**b**) r=0.15. (**c**) r=0.25. There are *N* curves in each panel, where one curve represents the opinion evolution of one agent.

Figure 2 shows each agent’s opinion $x_{i}\left(t\right)$ as a function of time *t* at α=1 (all agents are decentralized). Within finite time steps, the opinions in the system converge to clusters and no longer change with *t*; that is, the system achieves stability. When the system achieves stability, the opinion profile is fragmentation, polarization, and consensus at r=0.05, r=0.15, and r=0.25, respectively, similar to the classic HK model [16]. The final opinion profile can rely on different initial opinion configurations, but most work assumes a uniform distribution at random to keep it simple [21,22,23,24,25,26,27].

Below, we define that the system achieves stability at time *t* if $|x_{i}\left(t\right)-x_{i}\left(t-1\right)|<0.0001$, ∀i=1,2,⋯,N. We set the following statistical quantities to measure the system’s property at stability:

- $P_{C}$, the frequency of consensus in multiple runs ($0\leq P_{C}\leq 1$). In a run, if there is only one opinion cluster left in the system (e.g., Figure 2c), we say the system achieves consensus;
- $r_{1}$, the lower bounded confidence above which the system may consistently achieve consensus (i.e., $P_{C}<1$, $\forall r<r_{1}$, and $P_{C}=1$, $\exists r\geq r_{1}$). Similarly, $r_{0}$, the upper bounded confidence below which the system cannot achieve consensus (i.e., $P_{C}=0$, $\forall r<r_{0}$, and $P_{C}>0$, $\exists r\geq r_{0}$);
- $N_{C}$, the number of opinion clusters. For example, in Figure 2a–c, we have $N_{C}=7$, $N_{C}=2$ and $N_{C}=1$, respectively. $N_{C}=1$ means the system achieves consensus;
- $C_{\max}$, the relative size of the largest opinion cluster. We find the opinion cluster with the highest number of agents and divide it by *N*. Obviously, this quantity yields $1/N\leq C_{\max}\leq 1$;
- $\rho [x_{i}\left(T^{*}\right)]$, the distribution of stability opinions. We divide the range between 0 and 1 into 100 equal parts, and denote Δx=1/100=0.01. If $n\Delta x\leq x_{i}\left(T^{*}\right)<\left(n+1\right)\Delta x$, we add 1 to the distribution function at the *n*th part (n=1,2,⋯,100). After going through i=1,2,⋯,N, we divide the result in each part by *N*, and acquire the normalized opinion distribution;
- $T^{*}$, the convergence time. If $|x_{i}\left(t\right)-x_{i}\left(t-1\right)|<0.0001$, i=1,2,⋯,N, then, we denote $T^{*}=t$.

All the statistical quantities are the average over $10^{5}$ independent runs. Now, we study the system’s property at stability by these statistical quantities.

### 3.2. Results

**Figure 3:**
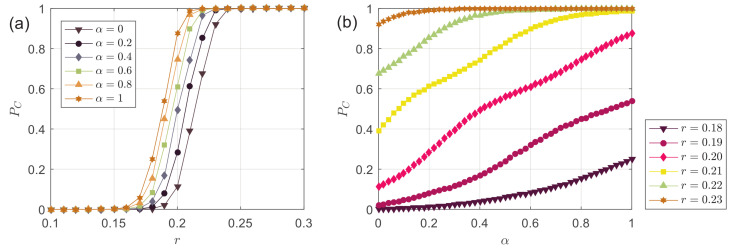
(**a**) The frequency of consensus $P_{C}$ as a function of the bounded confidence *r* at different α; (**b**) the frequency of consensus $P_{C}$ as a function of the fraction of decentralized agents α at different *r*.

In Figure 3, we study the frequency of consensus $P_{C}$. Figure 3a shows $P_{C}$ as a function of the bounded confidence *r* at different α. When α=0, the results are the same as the classic HK model. It is seen that as, *r* increases, $P_{C}$ gradually increases from 0 to 1 in the interval 0.15≲r≲0.25. The curves at different α show the same pattern. We can find a bounded confidence $r_{\alpha =1}\approx r_{\alpha =0}-0.03$ in the decentralized model (α=1) to reproduce the same $P_{C}$ value as the classic model (α=0). In other words, the larger the α, the larger the $P_{C}$ value of the corresponding curve at each point. To validate this, Figure 3b shows $P_{C}$ as a function of α at different *r* selected from the interval 0.15≲r≲0.25. It can be seen that $P_{C}$ always increases with an increase in α, which means the more decentralized agents in the system, the greater the frequency of complete consensus is.

**Figure 4:**
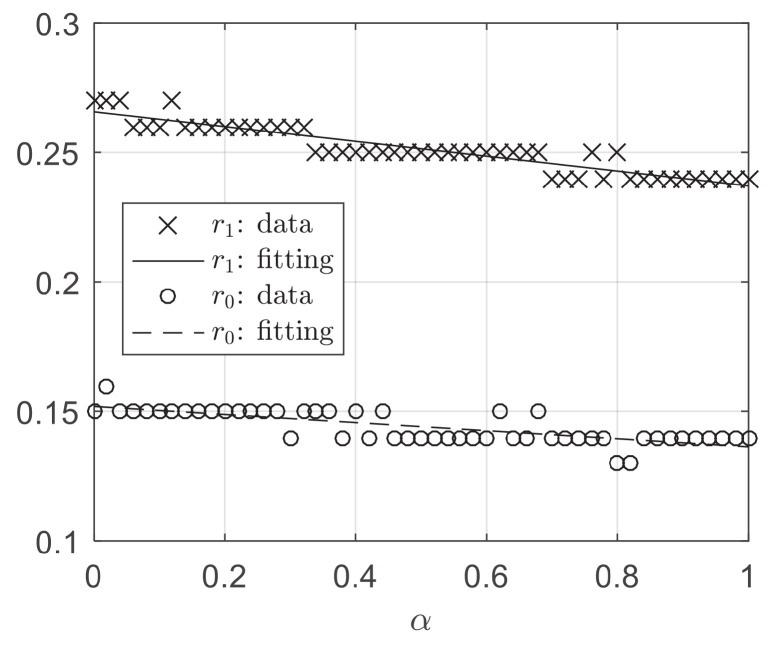
The lower bounded confidence $r_{1}$, above which the system may consistently achieve consensus (i.e., $P_{C}<1$, $\forall r<r_{1}$, and $P_{C}=1$, $\exists r\geq r_{1}$), as a function of α. The upper bounded confidence $r_{0}$, below which the system cannot achieve consensus (i.e., $P_{C}=0$, $\forall r<r_{0}$, and $P_{C}>0$, $\exists r\geq r_{0}$), as a function of α. The “data” derive from simulation, while the “fitting” derives from fitting a linear function to “data” using the least squares method.

A further approach to Figure 3 is studying the critical point where the opinion consensus emerges. Figure 4 shows the lower bounded confidence $r_{1}$ (above which the system may consistently achieve consensus) and the upper bounded confidence $r_{0}$ (below which the system cannot achieve consensus) as a function of α. Since the data points are scattered, a linear fit is performed to reveal the trend of the data. It is revealed that either $r_{0}$ or $r_{1}$ decreases with an increase in α. This illustrates that the larger the α, on the one hand, the earlier the $P_{C}$ starts to increase from 0 to 1, and, on the other hand, the earlier the $P_{C}$ ends the change from 0 to 1, finally reaching 1. Decentralized agents can advance the critical point of opinion consensus emergence.

**Figure 5:**
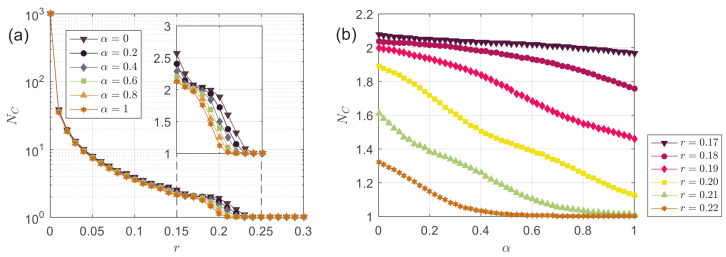
(**a**) The number of opinion clusters $N_{C}$ as a function of the bounded confidence *r* at different α; (**b**) the number of opinion clusters $N_{C}$ as a function of the fraction of decentralized agents α at different *r*.

More generally, we can study the final number of opinion clusters $N_{C}$ at the stationary state. Figure 5a demonstrates $N_{C}$ as a function of *r* at different α. Similar to the results of $P_{C}$, the function $N_{C}$ at different α share the same pattern. In particular, we can find an $r_{\alpha =1}\approx r_{\alpha =0}-0.03$ to reproduce the classic model when 0.15≲r≲0.25. As *r* increases, $N_{C}$ decreases, and the trend always presents a “steplike” behavior at different α. The breakpoints are distributed in 0.15≲r≲0.2, where “sharp steps” appear. The position of breakpoints is consistent with $r_{0}$ (see the panel inside Figure 5a), foretelling that opinion consensus will emerge as *r* continues to increase. In addition, we notice that the larger the α, the smaller the $N_{C}$ value of the corresponding curve at each point. Figure 5b further shows $N_{C}$ as a function of α at different *r*, which reveals that $N_{C}$ always decreases with an increase in α; that is, more decentralized agents lead to fewer opinion clusters in the system.

**Figure 6:**
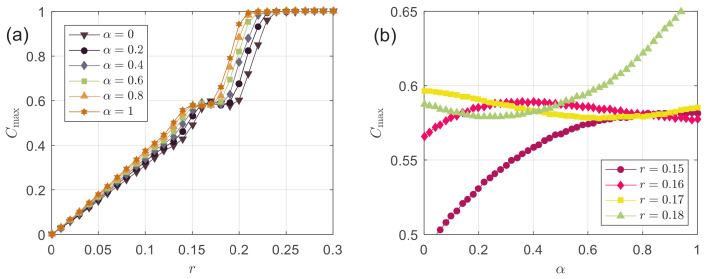
(**a**) The relative size of the largest opinion cluster $C_{\max}$ as a function of the bounded confidence *r* at different α; (**b**) the relative size of the largest opinion cluster $C_{\max}$ as a function of the fraction of decentralized agents α at different *r*.

Let us dig into more details. We show the relative size of the largest opinion cluster $C_{\max}$ as a function of *r* in Figure 6a. With an increase in *r*, the largest opinion cluster’s relative size $C_{\max}$ increases, indicating greater consensus in the system because more agents gather in the largest opinion cluster. At a larger α, the $C_{\max}$ value of the corresponding curve is greater; that is, decentralized agents facilitate the agents in the system to gather in the largest opinion cluster, forming opinion consensus. The “steplike” behavior can also be observed in the function $C_{\max}$, and the sharp steps appear in 0.15≲r≲0.2. The position of breakpoints is also consistent with those in Figure 5, where opinion consensus starts to emerge, implying that there is indeed a correlation between the relative size of the largest opinion cluster and the degree of opinion consensus. It is also worth noting that, in the “step-like” stage, α has non-monotonous effects on $C_{\max}$, as seen in Figure 6b, which is different from most situations observed in Figure 6a. Such non-trivial marginal phenomena may be worth exploring in the future.

**Figure 7:**
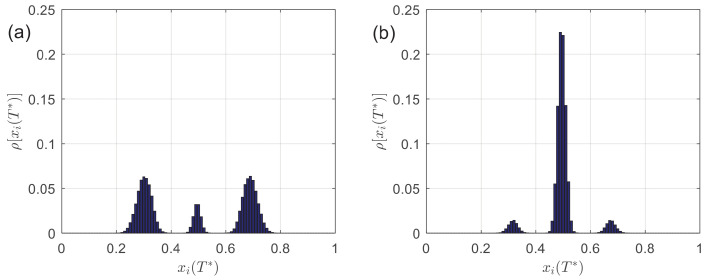
The distribution of stability opinions $\rho [x_{i}\left(T^{*}\right)]$ at r=0.2 and different α. (**a**) α=0; (**b**) α=1. The results are the average of $10^{5}$ independent runs.

Furthermore, Figure 7 presents the distribution of stability opinions $\rho [x_{i}\left(T^{*}\right)]$ at r=0.2, which provides more details than a relative size of the largest opinion cluster. In Figure 7a, α=0. From Figure 3 and Figure 5, we have $P_{C}\approx 0.11$ and $N_{C}\approx 1.89$. The distribution of stability opinions is mainly polarized, as shown on the two sides in Figure 7a. The consensus brings about the less central distribution reached cases. In Figure 7b, α=1. We have $P_{C}\approx 0.88$ and $N_{C}\approx 1.12$ from Figure 3 and Figure 5, respectively; opinion consensus takes the big lead. It can be seen from Figure 7b that the distribution on both sides is already sparse, and the opinions are mainly concentrated in the central area, $x_{i}\left(T^{*}\right)∼0.5$. Comparing Figure 7a,b, we say that more decentralized agents guide the stability opinions toward the central area in opinion space, promoting the opinion consensus.

**Figure 8:**
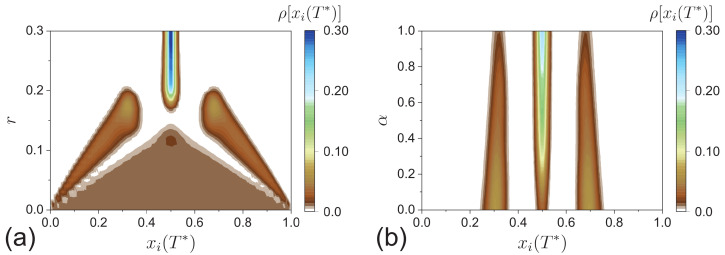
(**a**) The distribution of stability opinions $\rho [x_{i}\left(T^{*}\right)]$ as a function of the bounded confidence *r* at α=1; (**b**) the distribution of stability opinions $\rho [x_{i}\left(T^{*}\right)]$ as a function of the fraction of decentralized agents α at r=0.2.

Figure 8 shows the distribution of stability opinions $\rho [x_{i}\left(T^{*}\right)]$ as a function of specific parameters. The transverse profile in Figure 8 corresponding to a given vertical coordinate can be drawn in the form of Figure 7. Figure 8a shows $\rho [x_{i}\left(T^{*}\right)]$ as a function of *r* at α=1. As *r* increases, the system tends to consensus, and the stability opinions gradually concentrate towards the center area $x_{i}\left(T^{*}\right)∼0.5$ rather than an even distribution $0<x_{i}\left(T^{*}\right)<1$. At a qualitative level, though all agents are decentralized, the pattern in Figure 8a is the same as the classic HK model [16]. Figure 8b shows $\rho [x_{i}\left(T^{*}\right)]$ as a function of α at r=0.2, in which we can observe the process of decentralized agents promoting consensus. Consistent with Figure 7a,b, with an increase in α, the opinion distribution on the two sides gradually whitens, and the one in the central area fades to blue. The stability opinion profile transforms from polarization to consensus.

**Figure 9:**
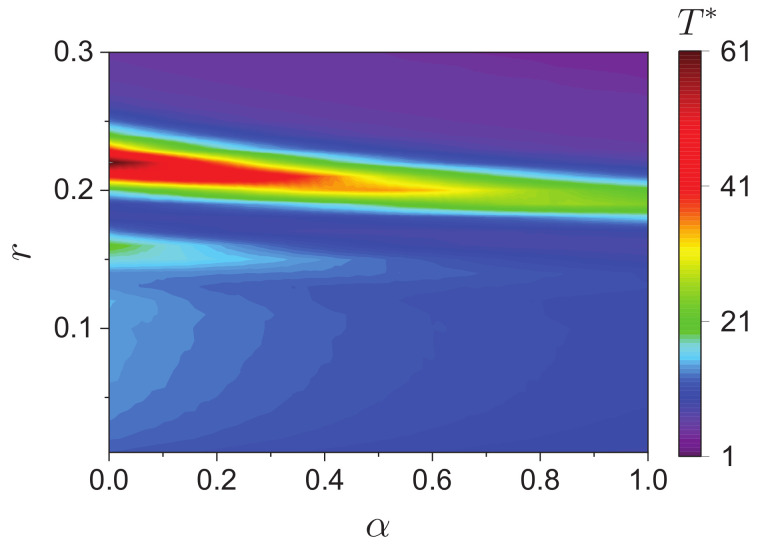
The convergence time $T^{*}$ as a binary function of the bounded confidence *r* and the fraction of decentralized agents α.

Finally, we study the convergence time $T^{*}$ as a binary function of *r* and α in Figure 9. The convergence time can also be used as a side measure of the role of decentralized agents on opinion consensus. It can be seen that the relatively time-consuming areas are two banded areas up and down. Looking at it vertically with *r*, the upper narrower band area corresponds to the region where $P_{C}$ increases from 0 to 1 in Figure 3. Looking horizontally at its variation with α, the narrower banded area gradually shifts downward as α increases, and its edges correspond qualitatively to $r_{0}$ and $r_{1}$ in Figure 4. This likewise indicates that the convergence time becomes larger in the process of consensus emergence (i.e., $0<P_{C}<1$). It is concluded from Figure 9 that, first, decentralized agents accelerate the convergence of opinions. Second, the variation pattern of $T^{*}$ with *r* does not change qualitatively with α.

### 3.3. Discussion

A further observation from Figure 9, however, indicates that the higher-order HK model at a specific bounded confidence $r_{\alpha =1}$ cannot be reached by simply rescaling $r_{\alpha =0}$ in the classic HK model—if we can find an $r_{\alpha =0}$ to reproduce the system behavior at an $r_{\alpha =1}$, then we should be able to observe an equal $T^{*}$ at α=1 to the one at α=0, which does not hold according to Figure 9. While the effective bounded confidence $r_{\alpha =1}\approx r_{\alpha =0}-0.03$ can be found for $P_{C}$ and $N_{C}$, the same operation is not practical for $T^{*}$. In particular, from opinion fragmentation to polarization, we cannot even find a rescaled $T^{*}$ between the two values of α.

The “decentralized” rule in this work can be seen as a linear superposition of the “centralized” one, as demonstrated in Section 2. It is common in complex systems that the simplest higher-order interaction can be seen as a linear transformation of the pairwise version. For example, the public goods game, which is the simplest multiplayer game that we mentioned in Section 1, can be understood as a superposition of the prisoner’s dilemma game [60] because of its linearity. The higher-order interactions, however, are not necessarily simple superpositions of pairwise interactions. An example is the *N*-person Hawk–Dove game, which reveals new phenomena compared with the 2-person version because of nonlinearity [61]. By analogy again, the higher-order bounded confidence model should potentially reveal non-trivial phenomena compared with the classic one once more complex rules are introduced. Even so, it should be noted that the simplest linear higher-order version is usually the most popular, especially when we study additional mechanisms other than the higher-order effect itself.

## 4. Conclusions

As an extension to the classic bounded confidence model where agents are influenced by peers through pairwise interactions, this paper introduced a possible framework of higher-order bounded confidence. The opinions of agents are influenced by group opinion discussions instead of by peers directly. The microscopic rule in each group discussion can be arbitrary, and we experimented with two underlying rules: centralized and decentralized. The former is equivalent to the classic HK model. From a series of statistic quantities, we showed that the decentralized rule, which represents a higher-order interaction compared with the centralized one, can promote opinion consensus and accelerate opinion convergence. Not surprisingly, the decentralized rule allows the interaction with opinions outside an agent’s original bounded confidence, which is somewhat equivalent to enlarging the bounded confidence despite such a transformation not always being effective or necessary.

However, the perspective of the model is more important than simply numerical results. In this work, the focal object for interactions is not agents, but rather groups. The group-based perspective to the classic bounded confidence model may bring the convenience of introducing other group-based dynamics into the bounded confidence model, such as the majority rule and other interdisciplinary dynamics. Since the function $o_{j}\left(t\right)=f\left(x_{k}\left(t\right)|k\in N_{j}\left(t\right)\right)$ determining the outcome of a single discussion is open-ended, the possible microscopic rules to be introduced are extensive.

To sum up, the “higher-order” interaction in this paper has two levels of inspiration. The first level is extending the first-order peers in opinion updating to the second-order, (i.e., the “decentralized” rule). The second level is to reconstruct the classic bounded-confidence model from the group-based perspective.

## Data Availability

The theoretical data used to support the findings of this study are already included in the article.
